# An Efficient and Geometric-Distortion-Free Binary Robust Local Feature

**DOI:** 10.3390/s19102315

**Published:** 2019-05-20

**Authors:** Jing-Ming Guo, Li-Ying Chang, Jiann-Der Lee

**Affiliations:** 1 Department of Electrical Engineering, National Taiwan University of Science and Technology, Taipei 10607, Taiwan; jmguo@seed.net.tw (J.-M.G.); russalchang@gmail.com (L.-Y.C.); 2 Department of Electrical Engineering, Chang Gung University, Tao-Yuan 33302, Taiwan; 3 Department of Neurosurgery, Chang Gung Memorial Hospital at LinKou, Tao-Yuan 33305, Taiwan; 4 Department of Electrical Engineering, Ming Chi University of Technology, New Taipei City 24301, Taiwan

**Keywords:** local invariant feature, feature detection, pattern matching

## Abstract

An efficient and geometric-distortion-free approach, namely the fast binary robust local feature (FBRLF), is proposed. The FBRLF searches the stable features from an image with the proposed multiscale adaptive and generic corner detection based on the accelerated segment test (MAGAST) to yield an optimum threshold value based on adaptive and generic corner detection based on the accelerated segment test (AGAST). To overcome the problem of image noise, the Gaussian template is applied, which is efficiently boosted by the adoption of an integral image. The feature matching is conducted by incorporating the voting mechanism and lookup table method to achieve a high accuracy with low computational complexity. The experimental results clearly demonstrate the superiority of the proposed method compared with the former schemes regarding local stable feature performance and processing efficiency.

## 1. Introduction

Nowadays, the feature point descriptor is one of the important topics in computer vision and machine learning and is being widely applied to image retrieval [1,2], object recognition [3,4], and even multi-view 3D reconstruction [5,6] or image stitching [7,8]. According to previous studies, Lowe [9] proposed the scale invariant feature transform (SIFT) algorithm in 2004, which integrates and optimizes steps to achieve a processing speed that is nearly real-time. In 2006, the study [10] revealed the speeded-up robust features (SURF) on the basis of the algorithm proposed by [9] to resolve the problem of how local stable features in the SIFT cannot be applied to real-time systems. In addition, Affine-SIFT [11] and Affine-SURF [12] have improved the rotation-invariance of SIFT and SURF. However, the two methods involve the use of floating-point descriptors which suffer from the issue of high complexity. Moreover, Alcantarilla et al. proposed KAZE features in 2012 [13] and then Accelerated-KAZE (A-KAZE) features in 2013 [14]. In contrast to the SIFT and SURF, which involve Gaussian image pyramids, these algorithms adopt nonlinear scale spaces to conduct multiscale analysis. In addition, the study [15] proposed the floating-point descriptor MROGH, which pools local features based on the intensity orders in multiple support regions. The calculation of A-KAZE and MROGH are still more complex than that of the SIFT and SURF.

To address the shortcomings of high complexity on floating-point descriptors, the study [16] in 2010 proposed the binary robust independent elementary features (BRIEF) to introduce the application of binary string vectors to local feature extraction. Subsequently, the study [17] in 2011 built on the features from accelerated segment test (FAST) to propose the oriented FAST and rotated BRIEF (ORB) method to resolve the problems of the rotation invariance and noise sensitivity of the BRIEF. The ORB accounts for the problem of image noise. Yet, it is not feature scale invariant, and it cannot resolve the issue of high complexity as the exhaustive search method is utilized to identify lowly correlated random points. In the same year, the study [18] proposed the binary robust invariant scalable keypoints (BRISK) to resolve the scale-space problem of the ORB. In 2012, the study [19] proposed a novel features descriptor, namely FREAK which related to human visual system to increase the accuracy. Although these methods of binary descriptors can achieve good efficiency, the issue of dissatisfactory matching performance remains unsolved because of the undesirable feature point selection. Consequently, several studies proposed the descriptor learning methods [20,21,22,23,24,25] which require to learn the optimal weighting through training processing to build the descriptors. Yet, the disadvantage of the above approaches is that these methods require high complexity when conducting the entire processing. On the other hand, Xianwei et al. [26] utilized ordinal and spatial information to provide a more discriminative description instead of comparing smoothed intensities, such as BRIEF, and adopted a cascade filtering design to speed up matching in 2014. Despite the fact that this method achieves better performance and shorter computation time, this method has a higher storage requirement than that of the BRIEF and ORB. The study [27] in 2016 proposed the binary descriptor with shared pixel blocks, namely BDSB, which divided a small number of scale-dependent patches into overlapping blocks of pixels to accelerate the processing, but this method is still of low matching performance. Finally, in 2018, the novel binary descriptor based on binary weights learning approach was proposed which refers to as a robust weighted binary descriptor (RWBD) [28] and this method can be categorized into the descriptor learning method.

This paper contributes to realizing an innovative local stable feature method on the basis of the binary descriptor instead of the float-point descriptor method and without the descriptor learning technique. Moreover, the proposed method can achieve lower complexity, enhanced accuracy, as well as robust scale, rotation, and lighting invariance. Section 2 elucidates various aspects of the fast binary robust local feature (FBRLF) algorithm proposed in this study, including scale-space feature detection, feature point localization, feature point orientation assignment, feature point descriptor, and feature point matching. In addition, to reduce computation time, the proposed FBRLF algorithm does not need any learning procedure. Section 3 mainly focuses on local stable features obtained using the proposed FBRLF algorithm, and other methods under various photographing conditions were analyzed and matched. Finally, Section 4 concludes this study.

## 2. Materials and Methods

**Figure 1:**
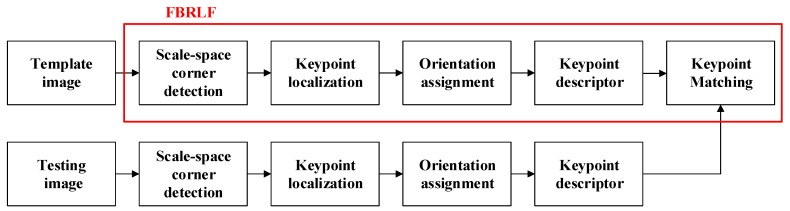
A flow chart indicates the template image and testing image are both utilized the proposed FBRLF method to compare the similarity. The procedure of the proposed FBRLF method is shown as the red box.

This paper proposes a highly effective local stable feature technique, namely the FBRLF algorithm. In contrast to the former methods, this algorithm exhibits good invariance tolerance against scaling, rotation, and lighting chances, as well as enabling high-efficiency processing. The FBRLF first applies multiscale adaptive and generic corner detection based on the accelerated segment test (MAGAST) detector to extract stable feature points. Subsequently, a two-octave image pyramid is established to maintain scale-space invariance. To ensure that feature vectors are rotation-invariant, the FBRLF does not adopt a main orientation as that used in the SIFT and SURF. Instead, the proposed algorithm applies a fixed image rotation approach to ensure the rotation invariance of the feature points. In addition, binary strings established through the BRIEF are used as feature descriptors of the FBRLF. Finally, the proposed algorithm involves a voting mechanism to reduce the processing time of feature matching. Figure 1 illustrates the overall process of the FBRLF.

### 2.1. Scale-Space Feature Detection

The accelerated segment test (FAST) [29] is a famous corner feature point detection, and the adaptive and generic corner detection based on the accelerated segment test (AGAST) [30] can significantly improve the FAST algorithm on its performance. Yet, feature points acquired through the FAST and AGAST are not scale-invariant. Consequently, this study proposes the multiscale AGAST (MAGAST) detector, which adopts a two-octave image-capturing technique to maintain scale-space invariance and proposes adaptive optimum threshold value based on AGAST which underlies the FAST. Specifically, this technique involves sampling an image twice, and prevents extensive calculation. Thus, it can achieve multiscale invariance, and increase the overall system efficiency. First, a set of captured images is processed for each octave. The first-octave and second-octave image sets are denoted as $\left\{L_{0},L_{1},L_{2},\ldots L_{n-1}\right\}$ and $\left\{M_{0},M_{1},M_{2},\ldots M_{n-2}\right\}$, respectively. The process for building a two-octave image pyramid is described as follows.

(1) If $L_{0}$ is the original image undergone smoothing once, then $L_{1}$ is generated by decimating $L_{0}$ by 1/2, $L_{2}$ is generated by decimating $L_{1}$ by 1/2, and so on. These images form the first-octave image set.
(2) The second-octave image set is established on the basis of the first-octave image set. Specifically, M_0_ is generated by decimating $L_{0}$ by 2/N, where N is the number of images per octave. *M*_1_ is generated by decimating $M_{0}$ by 1/2, *M*_2_ is generated by decimating $M_{1}$ by 1/2, and so on.

**Figure 2:**
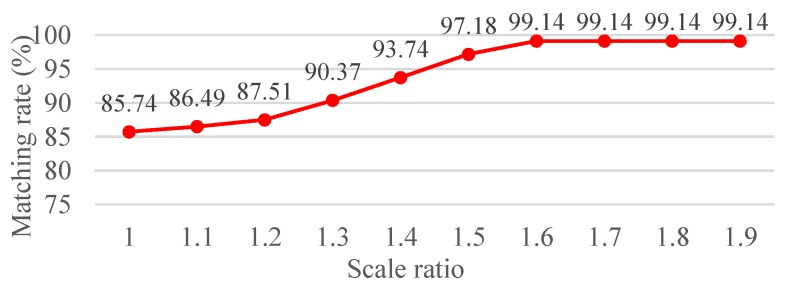
Matching rate under various scale ratio values.

In Equations (1) and (2), $S\left(L_{i}\right)$ and $S\left(M_{i}\right)$ represent the scale relationships of the first-octave image L*_i_* and second-octave image M*_i_*, respectively.

(1) $$S\left(L_{i}\right)=2^{i},$$

(2) $$S\left(M_{i}\right)=2^{i}\times K,$$

where K is the scale ratio, which is conducted by the experimental result of the matching rate, and the result is the average value obtained over 100 natural images of size 800 × 600. Thus, in this study, the parameter K is set at 1.6 since it can achieve the same performance after scale ratio 1.6 as shown in Figure 2. Finally, the AGAST algorithm is conducted in the dispersed scale space represented to determine the candidate region of a feature. Although, a high number of scales facilitated the detection of additional feature points, these feature points were highly unstable, thus reducing feature repeatability.

**Figure 3:**
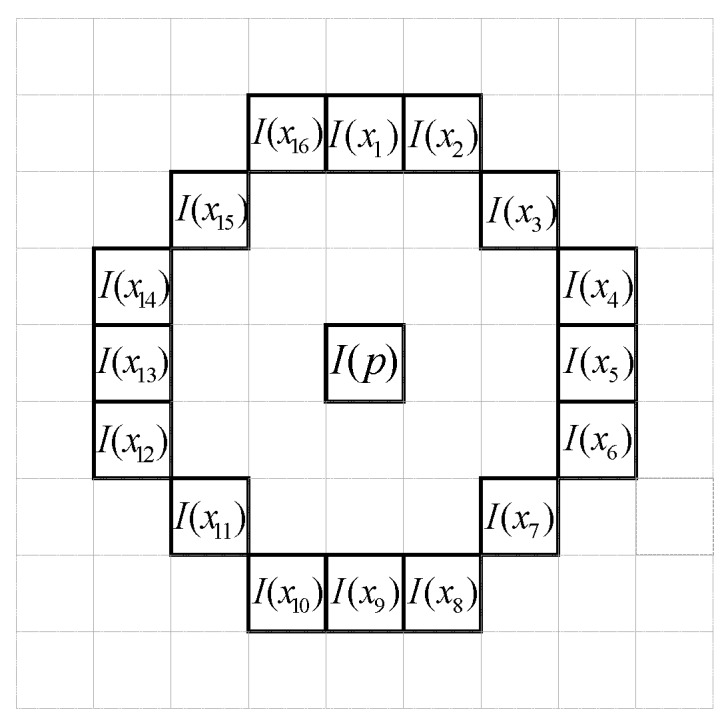
Sixteen pixels I(x) to examine whether I(p) is the feature point or not.

According to the aforementioned problems, this paper proposes the MAGAST feature detection method, in which the average number of feature points detected through the AGAST is used as the threshold value. This is advantageous for determining the optimum threshold value for each image to effectively increase the scale-space feature repeatability. In addition, this method involves a bit shift technique to reduce the time required for the overall calculation as well as for determining the optimum threshold value. The process of the MAGAST is described as follows:

(1) The lower and upper bound parameters of an image are defined under different scales. More details for the lower and upper bound parameters will be presented in Section 2.1.1.
(2) The AGAST feature detection method is applied to the image, and the number of identified feature points is determined as
  (3) $$N_{1}=\sum_{\forall x\in (circle\left(p\right))}\left\{1\;|\;\left|I\left(i,j\right)+T\right|>I\left(x\right)\right\},$$
  (4) $$N_{2}=\sum_{\forall x\in (circle\left(p\right))}\left\{1\;|\;\left|I\left(i,j\right)-T\right|>I\left(x\right)\right\},$$
  (5) $$N=\sum \left[\underset{\left(i,j\right)}{\sum }\left\{1\;|\;\left(N_{1}⨂N_{2}\right)>\;TC\times \frac{3}{4}\right\}\right],$$
  where I(x) is the grayscale value of a random point on the circumference of the circle as shown in Figure 3. The variable I(p) is the center’s gray scale value. The variables T and TC represent the threshold of the grayscale value and the total number of circles, respectively. The parameters, (i,j) and ⨂, are the location of potential corner points in the image and OR operation, respectively. Assuming that two consecutive 3/4 of the $N_{1}$ or $N_{2}$ values are greater or smaller than the threshold, p is then confirmed as a corner feature in the target image. Thus, N denotes the number of feature points. In addition, the AGAST feature detection method has speeded up the FAST algorithm on the accelerated segment test.
(3) If the number of feature points is lower than the lower bound or higher than the upper bound, then the threshold value is adjusted.
  - If the number of detected feature points is less than the lower bound, then the threshold value is too rigorous, preventing valid feature points from being detected. Thus, the threshold value is reduced to increase the number of feature points by using Equation (6):
    (6) $$T^{\prime }=\left(T+Left\right)\gg 1$$
  - If the number of detected feature points exceeds the upper bound, then the threshold value is too lenient, and numerous unstable feature points will be detected. Consequently, the threshold value is increased to reduce the number of feature points by using Equation (7):
    (7) $$T^{\prime }=\left(T+Right\right)\gg 1$$
    T is the unadjusted threshold value, and $T^{\prime }$ is the adjusted threshold value. Left and Right are dynamic parameters that change along with each threshold value adjustment. These two parameters are primarily used to increase or reduce the threshold value, thus increasing or reducing the number of feature points. The symbol ≫ is a bitwise right shift operator.
(4) When adjusting the threshold value $T^{\prime }$, the optimum threshold value is identified when Equation (8) is met.
  (8) $$T^{\prime }=Right\;or\;T^{\prime }=Left$$
  Equation (8) indicates that the new threshold value has been used as the adjusted threshold value during the adjustment of the lower bound or upper bound. In other words, the adjustment results in a repeated threshold value, which represents the optimum threshold value. If the optimum threshold value is not found, then Steps 2 and 3 are repeated until it is identified and the pseudo code of the MAGAST is detailed as shown in Algorithm 1.

**Algorithm 1 Pseudo code**—Multiscale AGAST (MAGAST) Detection.
**1: BEGIN**
**2:** Input image, Lower Bound and Upper Bound.**3:** Set Left = 0, Right = 255, Threshold = 128, Score = 256;**4: WHILE** (Score < Lower Bound || Score > Upper Bound)**5:** Score = use Threshold to Detect AGAST features on image**6: IF** (Lower Bound > Score)**7:**  Right = Threshold**8:**  Threshold = (Threshold + Left) >>1**9:  IF** (Right == Threshold)
**10: END WHILE**

**11:  END IF**

**12: ELSE**
**13:**  Left = Threshold**14:**  Threshold = (Threshold + Right) >>1**15:  IF** (Left == Threshold)
**16:   END WHILE**

**17:   END IF**

**18: END**


Finally, the simple example is used to elucidate the aforementioned procedure (Table 1), and the following assumptions are made: The parameters Left, Right, Threshold, Score, Lower Bound (LB) and Upper Bound (UB) are set at 0, 8, 4, 256, 8, 198, respectively. In the first step, the AGAST method detects the higher feature points (500) than the value of UB (198), leading to increasing the value of Threshold. After several round of procedures, the best value of threshold (7) is found at Line 13 in step 3 because the value of Left and Threshold are matched, and thus finish the MAGAST operation.

**Figure 4:**
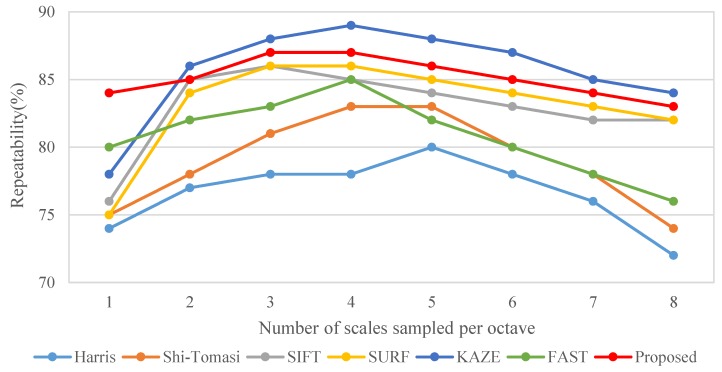
Performance comparison between MAGAST (proposed) and other feature detectors with relationship between the number of scales and repeatability.

**Figure 5:**
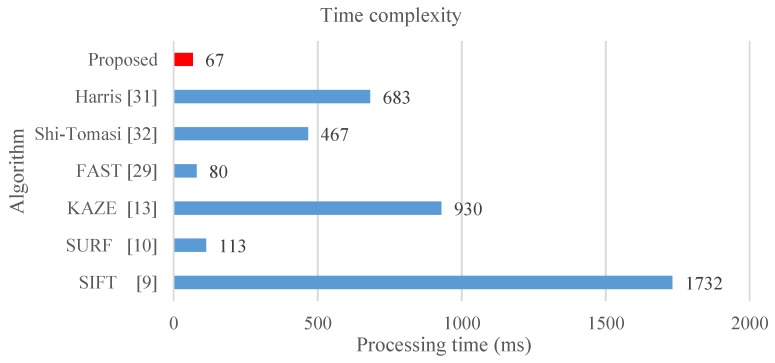
Performance comparison between MAGAST (proposed) and other feature detectors with relationship between the three of scales and processing time. The images are of size 800 × 600 and approximately 1000 keypoints are detected.

Figure 4 presents the effects of the MAGAST, which is applied to a scale space that is established through the two-octave capturing technique and the other detection algorithms, including SIFT, SURF, KAZE and FAST, and its effect on the relationship between the number of scales per octave and feature repeatability. The repeatability is defined in [9]. In addition, the popular corner detectors are also investigated such as Harris [31] and Shi-Tomasi [32]. The results are average values obtained through 100 natural images (800 × 600). The results revealed that the proposed MAGAST features effectively increased the feature repeatability, thereby enhancing the matching rate in the subsequent feature matching. The matching rate is defined as the number of correctly matched feature points and the number of falsely matched feature points obtained between the standard image and the testing image. Moreover, the MAGAST can achieve a higher repeatability under a low scale space (n = 3) compared to that of the Harris (n = 5), Shi-Tomasi (n = 4), SIFT (n = 3), SURF (n = 3) and FAST (n = 4). Finally, although KAZE can achieve the best performance in scale space (n = 4), this method has higher complexity than that of the proposed method as shown in Figure 5.

#### 2.1.1. Lower Bound and Upper Bound Parameters

The MAGAST feature detection algorithm applies the lower bound and upper bound parameters to automatically adjust the threshold value for detecting features, thereby increasing the repeatability of the features identified in a scale space. To enhance the solutions of the lower and upper bound parameters, a statistical concept is applied to solve the optimum parameters.

The lower bound and upper bound parameters pertain to a mechanism for controlling the feature number range and for determining the most appropriate feature detection threshold value. In this paper, a statistical method is used to determine the lower bound and upper bound parameters. Specifically, feature points in 1000 natural grayscale images are detected to examine on natural images, and the average number of detected feature points is applied as the basis for the parameter’s determination. In addition, the process of training lower bound and upper bound parameters utilizes the AGAST [18] as the ground truth. On the other hand, image size is also a key factor when building the scale space. Because a scale space shrinks an image, the number of detectable feature points is also reduced. Thus, in this study, six groups of images as shown in Table 2 with various sizes were utilized as the testing samples.

Table 2 illustrates that the image size ranges from 160 × 120 to 1280 × 960. The 1000 natural grayscale images in each group were used to conduct a statistical analysis. Feature detection was applied to each image group, and the number of features detected in each image was recorded. Finally, the numbers of detected features with the 1000 natural images were averaged to determine the lower bound and upper bound parameters of each image group as shown in Table 3.

### 2.2. Keypoint Localization

After feature points are obtained using the FBRLF, methods similar to the SIFT [9] and BRISK [18] can be used to determine the feature point information, including location, scale, and curvature. Subsequently, low contrast and unstable feature points are removed, and the remaining candidate feature points are also called keypoints.

**Figure 6:**
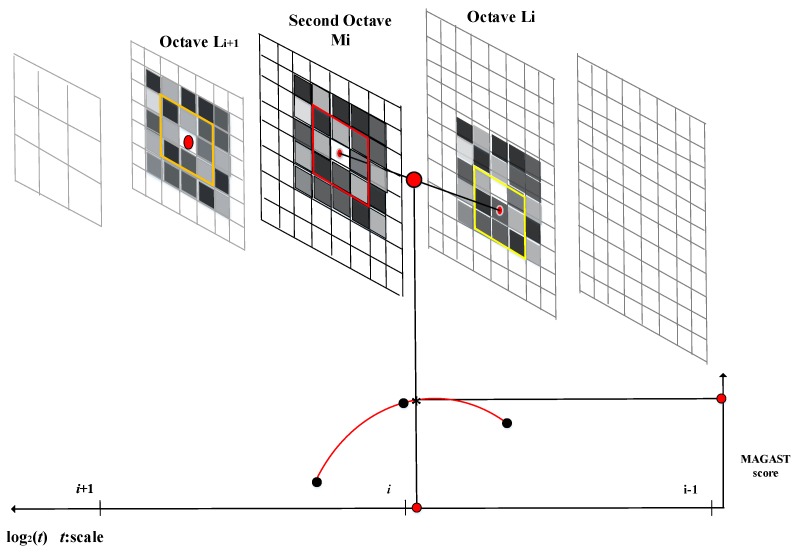
Maximum and minimum values determination of the candidate feature.

To identify the most stable point, i.e., the minimum or maximum point, in a scale space, each sampling pixel point is compared with its neighboring pixel points to determine whether it is larger or smaller than the neighboring points in its spatial domain. After the two-octave capturing technique is employed to construct an image pyramid, each image in the pyramid is tested using the MAGAST corner detection method to determine the candidate regions. Subsequently, the non-maximum suppression is applied to the 3 × 3 cubic region formed by the spatial domain and neighboring scales of each candidate region to determine the stable feature point in this region. Figure 6 illustrates this multiscale feature detection scheme. Each extremum point localization process involves using the first-octave and second-octave images as the judgement and reference layers, respectively. A 3 × 3 cubic region is defined around each sampling point (feature) in the candidate regions of the second-octave image. The upper and lower layers of the cubic region are equivalent to the upper and lower layers of the first-octave image (Li and Li+1). Subsequently, a cross-reference search method is applied to determine the coordinates and scale of the extremum point. After the extremum point in the first second-octave image is identified, the next second-octave image is used as the reference layer. Accordingly, Li+1 and Li+2 are used as the upper and lower layers of the 3 × 3 cubic region, respectively, and so on. For example, for image Mi, the candidate features and its eight neighboring pixel points are selected, the red block and their MAGAST response values are compared. In addition, the MAGAST response value of the candidate feature is also compared with those of the nine pixels in the neighboring first-octave upper layer (Li) as the yellow block and those of the nine pixels in the neighboring first-octave lower layer (Li+1) as the orange block. This enables the determination of whether the candidate feature contains the extremum value of the local MAGAST response value. After the non-maximum suppression, a parabolic function is fitted in the scale and image spaces. The maximum point of this function corresponds to the accurate scale of the feature. Finally, a quadratic interpolation method is used to determine the exact location of the feature point. In other words, the four neighboring pixels are employed as the subpixels. The coordinates of the subpixels are adjusted to determine the accurate location of the maximum or minimum point.

### 2.3. Orientation Assignment

The FBRLF algorithm can be used to obtain rotation-invariant features by fixating feature point rotation at various angles. The feature points of an image can be identified through a scale space to achieve scale invariance. Subsequently, the image is rotated to different angles to achieve rotation invariance. During the calculation, the image location can exhibit rotation, scaling, and translation effects. Applying scaling, rotation, and translation transformation to each image increases the overall time complexity of the FBRLF. Consequently, this study applied the affine transformation method, through which linear combination is used to integrate various transformation processes into a single matrix. This method is also called composite transformation.

After applying MAGAST feature detection, all of the detected feature points are then undergone translation transformation, followed by scaling and rotation transformation. As the scaling and rotation transformation is completed, another translation transformation is conducted to convert all feature points back to their original coordinates as formulated below.

(9) $$\overset{\mathrm{Translation}}{\left[\begin{array}{ccc} 1 & 0 & center.x\\ 0 & 1 & center.y\\ 0 & 0 & 1 \end{array}\right]}\overset{\mathrm{Scaling}}{\left[\begin{array}{ccc} s & 0 & 0\\ 0 & s & 0\\ 0 & 0 & 1 \end{array}\right]}\overset{\mathrm{Rotation}}{\left[\begin{array}{ccc} \cos\left(\theta \right) & -sin\left(\theta \right) & 0\\ sin\left(\theta \right) & \cos\left(\theta \right) & 0\\ 0 & 0 & 1 \end{array}\right]}\overset{\mathrm{Translation}}{\left[\begin{array}{ccc} 1 & 0 & -center.x\\ 0 & 1 & -center.y\\ 0 & 0 & 1 \end{array}\right]}$$

where center.x and center.y represent the coordinates of the image center; s and θ indicate the scale factor and angle of rotation, respectively. More details for the θ parameter will be presented in Section 2.5.2. Next, matrices in (9) are multiplied to obtain (10).

(10) $$\left[\begin{array}{ccc} scos\left(\theta \right) & scos\left(\theta \right) & \left(1-scos\left(\theta \right)\right)center.x-scos\left(\theta \right)center.y\\ -scos\left(\theta \right) & scos\left(\theta \right) & ssin\left(\theta \right)center.y+\left(1-scos\left(\theta \right)\right)center.y\\ 0 & 0 & 1 \end{array}\right]$$

**Figure 7:**
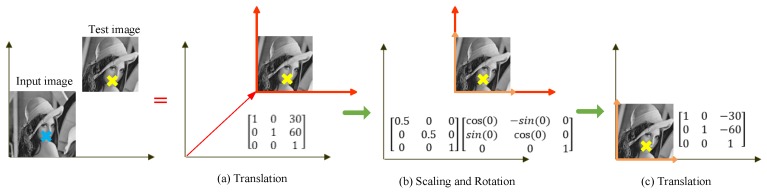
Illustration of the FBRLF orientation assignment.

Equation (10) is essential to the rotation invariance of the FBRLF. This equation enables the calculation of scaling and rotation transformation using a single matrix; thus, the resulting image accounts for scaling and rotation variations and reduces the calculation time through processing multiple matrices. Subsequently, sample images are used to illustrate the Equation (9) as shown in Figure 7. The “X” marked in blue and yellow indicates the coordinates of the feature point on the input and test image, respectively, and it is expected to convert the yellow mark “X” into the corresponding coordinate of the blue mark. First, the coordinate of the input image needs to translate the coordinate to the test image. Assuming the displacement is (30, 60), the matrix representation is shown in Figure 7a. Second, comparing to the input image, the test image has the scale change (assuming 0.5 times of the original), leading to modifying for the red coordinate to the orange coordinate. However, the test image does not have the rotation change (θ=0), and thus maintain the orange coordinate as shown in Figure 7b. Finally, after applying translation, scaling and rotation transformation, the yellow mark “X” adopts another translation to return its original coordinate as shown in Figure 7c.

### 2.4. Keypoint Descriptor

An ideal feature description vector should be highly robust and distinctive. Matching speed, which is also an essential factor, refers to the processing and computation capability for comparing the similarity between two sets of feature points. A high feature dimension results in a low matching speed.

According to the aforementioned assertions, the FBRLF is applicable for resolving associated problems. The FBRLF uses the BRIEF [16] as the feature descriptor. The BRIEF applies binary data structure information, and is advantageous because of its fast matching speed. However, this method is easily affected by noise. The BRISK [18] in an alternative that adopts dissimilar Gaussian core models to revolve the noise interference of the BRIEF. However, using various Gaussian core models to process noise around each feature point is time-consuming. Consequently, this study proposes the Gaussian weighting approach to conduct templatization. In addition, the integral image approach [33] is adopted to reduce processing time and thus control noise.

**Figure 8:**
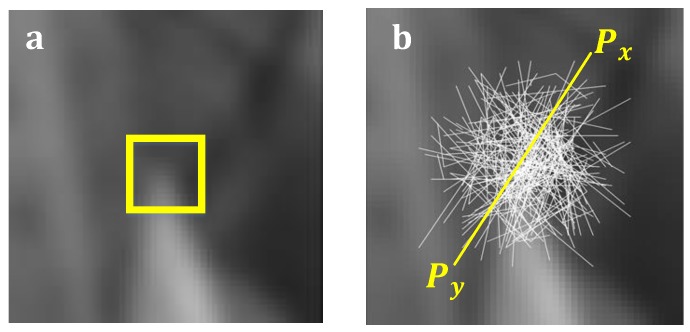
(**a**) A feature point on image; (**b**) Put a patch on feature point.

The feature point description process of the FBRLF involves a concept similar to that of the BRIEF. The parameters of the BRIEF used in this study accord to those provided by the author of the BRIEF. The BRIEF algorithm is advantageous because of its fast processing speed, but it is also highly sensitive to noise. The processing steps are as follows:

First, FBRLF defines test τ on patch p of size S × S as

(11) $$\tau \left(p;x,y\right)≔\{\begin{array}{ll} 1 & if\;P_{x}<P_{y};\\ 0 & otherwise, \end{array}$$

where $P_{x}$ and $P_{y}$ are the pixel values in end points of the lines and each of the lines are come from the patch p as shown in Figure 8.

**Figure 9:**
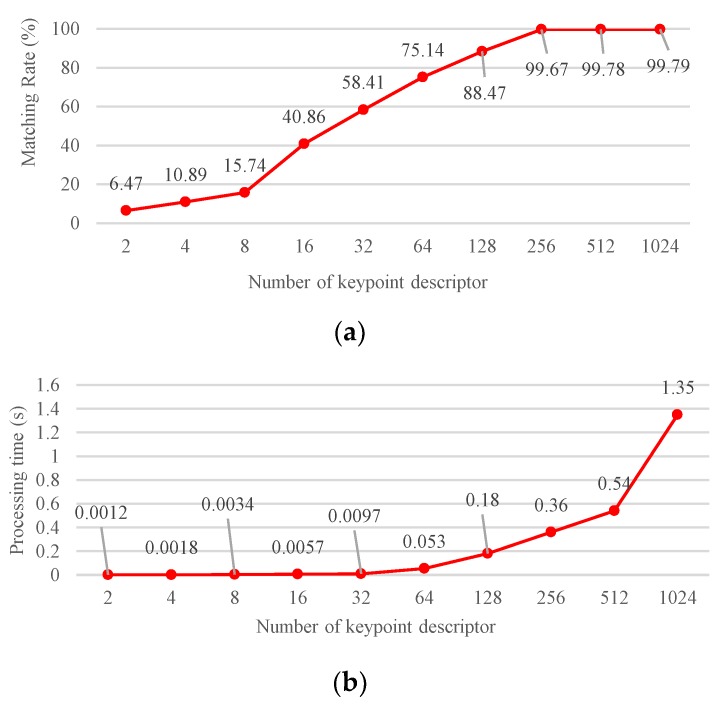
Performance comparison between (**a**) matching rate and (**b**) processing time with various numbers of keypoint descriptors.

Second, FBRLF take BRIEF descriptor to be the bit string as Equation (12)

(12) $$f_{n_{d}}\left(p\right)=\underset{1\leq i\leq n_{d}}{\sum }2^{i-1}\tau \left(p;x_{i},y_{i}\right)$$

where the parameter $f_{n_{d}}$ is the number of keypoint descriptors as shown Figure 9. To achieve high efficacy performance, this study sets the parameter $f_{n_{d}}$ at 256.

Finally, to resolve the noise problem, this study proposes applying a Gaussian weight template to $P_{x}$ and $P_{y}$ coordinates using Equation (13):

(13) $$\beta \left(i,j\right)≔\{\begin{array}{ll} 1 & if\;P_{x}^{\prime }>P_{y}^{\prime };\\ 0 & otherwise, \end{array}$$

where $P_{x}^{\prime }$ and $P_{y}^{\prime }$ represent $P_{x}$ and $P_{y}$ processed using the Gaussian weight template; β(i,j) denotes the final comparison outcome. Equation (13) indicates that the feature descriptor of the FBRLF is constructed through a binary method.

**Figure 10:**
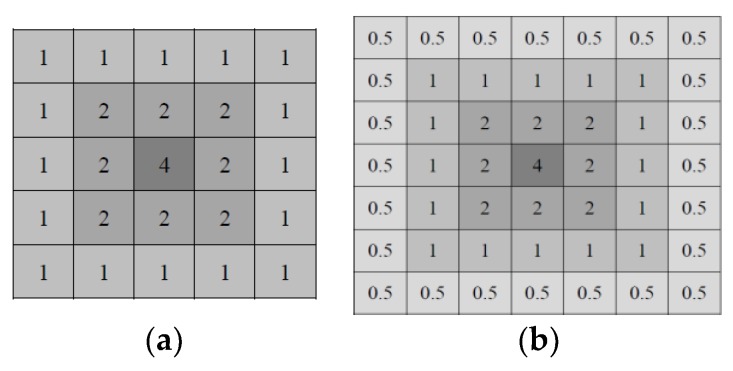
Gaussian weighting templates. (**a**) 5 × 5 and (**b**) 7 × 7.

A Gaussian filter is a type of low-pass filter derived from the Gaussian probability distribution. The values near the center of the Gaussian are large, and the values decrease as their distance from the center increases. Similarly, when examining the relationship of a feature point and its surrounding pixels, the pixels near the feature point more strongly influence the feature point than do those that are far from it. Using a conventional method to calculate a 4 × 4 Gaussian filter involves 16 multiplication and 16 addition operations, and a large filter requires additional calculation processes. For this problem, this study proposes using Gaussian filters of various sizes to process box filtering and weighting. Relevant equations are described as follows:

(14) $$P_{x}^{\prime }\left(i,j\right)=P_{x}\left(i,j\right)*GW\left(s,t\right)$$

(15) $$P_{y}^{\prime }\left(i,j\right)=P_{y}\left(i,j\right)*GW\left(s,t\right)$$

where $P_{x}^{\prime }\left(i,j\right)$ and $P_{y}^{\prime }\left(i,j\right)$ are grayscale values after utilizing a Gaussian weight template; GW denotes the Gaussian weight template; (i,j) and (s,t) represent the positions of the feature point and the Gaussian weight template, respectively, and the operation ∗ denotes convolution. Figure 10 shows a 5 × 5 and a 7 × 7 Gaussian weighting templates.

**Figure 11:**
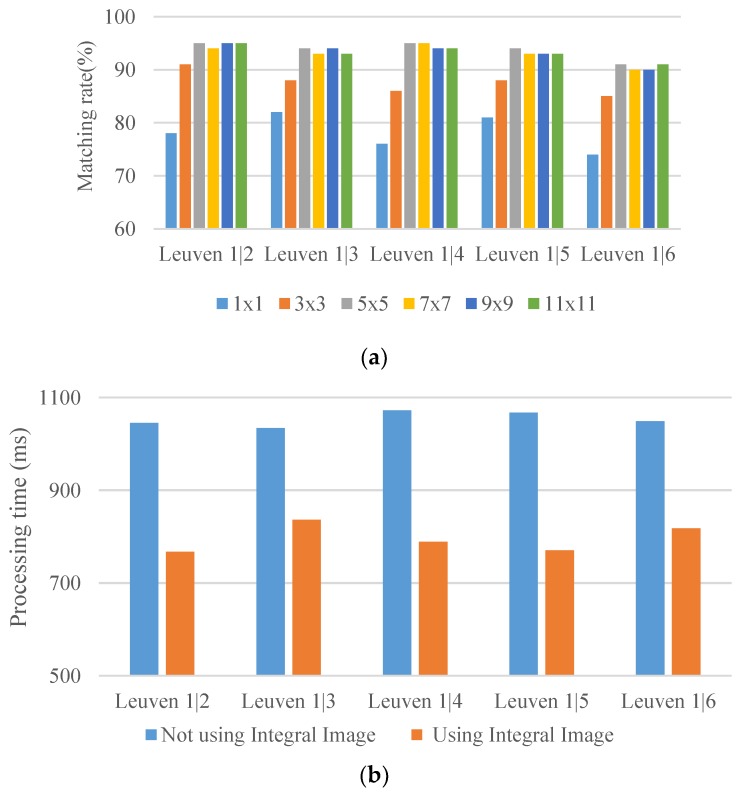
Matching rate and calculation time comparison between with and without the use of integral image. (**a**) Matching rate achieved through Gaussian weighting templates of various sizes; (**b**) Calculation time required for the Gaussian weighting templates when the integral image is applied.

Figure 11a illustrates the results of applying dissimilar Gaussian weighting templates to a public database [34] under the Leuven database. When a large Gaussian filter is used, the values that are far from the feature center are small and approximately 0 at the boundary of the filter. The results revealed that the matching rate acquired using the 5 × 5 Gaussian weighting template was higher than that obtained using the 7 × 7 Gaussian weight template. To reduce the calculation time, this study applied the 5 × 5 Gaussian weight template. In addition, to improve the processing efficiency, the integral image representation was used to increase the spatial filtering of the Gaussian weight template as Figure 11b; this is also a key reason why the Gaussian weightings are templatized.

**Figure 12:**
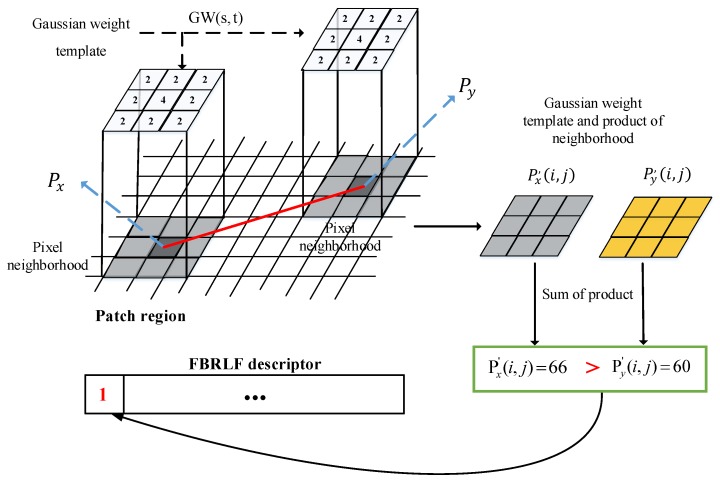
FBRLF feature description. The red line is one of the lines from the patch.

Finally, sample images are used to elucidate the aforementioned procedure (Figure 12). In an input image, the proposed algorithm is adopted to identify a robust and stable feature point. Subsequently, the patch region around the feature point and the proposed Gaussian weighting template approach are employed for calculation. The speed is further accelerated using the integral image approach [33]. Thus, the noise effect can be well handled, and the computation time can be reduced as well. Subsequently, the aforementioned procedure is used to calculate and compare with $P_{x}^{\prime }\left(i,j\right)$ and $P_{y}^{\prime }\left(i,j\right)$ which is one of the end points of the lines from the patch. The final comparison outcome is then fed into the FBRLF feature descriptor as described in 1 because the value of $P_{x}^{\prime }\left(i,j\right)$ is greater than $P_{y}^{\prime }\left(i,j\right)$. In the end, the FBRLF descriptor is completely created when all of the lines are finished comparison in the patch.

### 2.5. Keypoint Matching

The feature matching technique adopted by the proposed FBRLF integrates the exhaustive and indexing methods. When assigning the orientations of the feature points, the FBRLF establishes a feature index that accounts for various angles. However, the proposed algorithm cannot be used to determine the rotation difference between two images; thus, the exhaustive method is applied to analyze the feature set under each angle, thereby determining the optimum angle difference. However, this method incurs additional calculation time when the size of the feature index and the number of feature points increase. Under an extreme scenario, the exhaustive method prevents real-time calculation. Consequently, this study proposes a voting mechanism and lookup table method to resolve the aforementioned problem and effectively enhance the overall calculation performance. Because computing costs feature vectors acquired through the FBRLF are binary data, Hamming distance can be used to match the distance vectors. Moreover, matching binary data is faster than matching float-point values.

#### 2.5.1. Hamming Distance

In this study, if the Hamming distance between the FBRLF description of the sample feature and that of the database feature exceed $T_{HD}$, then the two features are not matched. Otherwise, the two features are matched.

(16) $$M=\{\begin{array}{cc} 1 & if\;Hamming\;distance<T_{HD};\\ 0 & otherwise, \end{array}$$

where M denotes whether two features are matched. If the Hamming distance is less than 40, then the two features are considered as matched (M = 1). Otherwise, M = 0, indicating that the two features are not matched.

**Figure 13:**
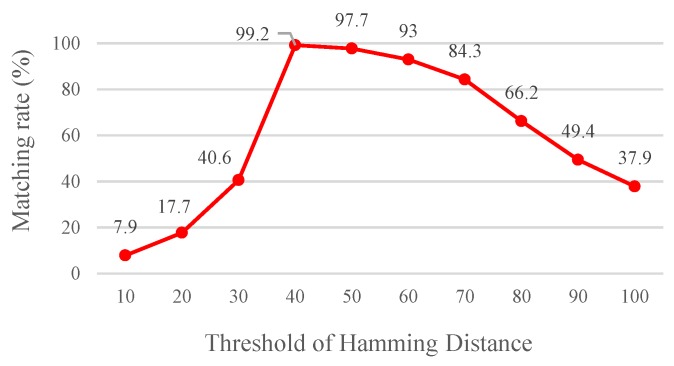
Matching rate under various Hamming distance threshold values.

**Figure 14:**
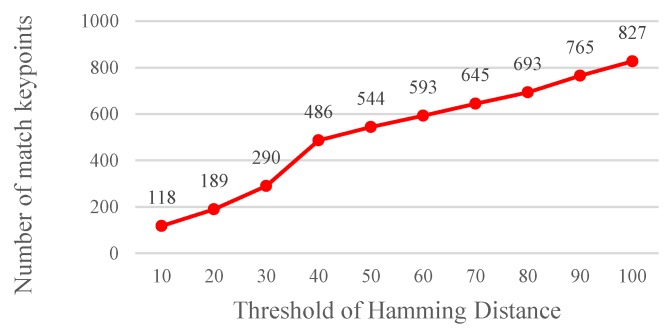
Relationship between the Hamming distance threshold value and number of matched keypoints.

A Hamming distance <$T_{HD}$, which was used as the basis for matching feature points, was determined through an experiment. Using the procedure mentioned in Section 2.1.1, it shows that the optimum lower bound and upper bound parameters can be obtained. The results show that the matching rate is the highest when the Hamming distance threshold value $T_{HD}$ is set at 40 (Figure 13). A high Hamming distance threshold indicates a high tolerance value. Moreover, the threshold value is directly correlated with the number of matched images (Figure 14). Yet, more matched feature points do not imply a desired matching outcome since the matched feature points might be unstable, indicating that a high tolerance value is associated with a high probability of erroneous feature point matching.

#### 2.5.2. Lookup Table Method and Voting Mechanism

**Figure 15:**
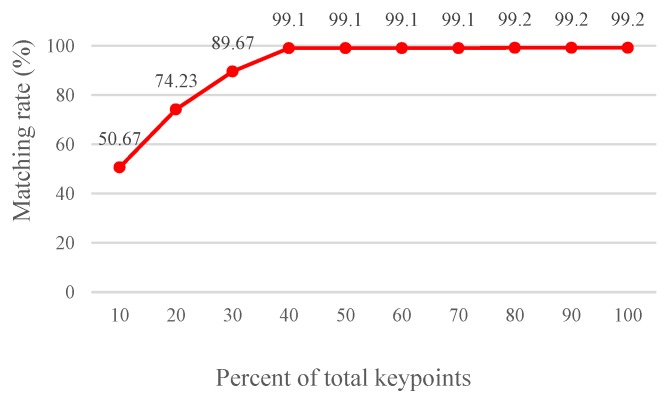
Matching rate achieved using various percentage ratios of features.

This method requires constructing a feature lookup table. The feature points detected through the scale-space and localization processes are categorized according to their rotational angles. Equation (17) illustrates the format of the lookup table:

(17) $$\left(\begin{array}{c} \begin{array}{ccc} \theta_{1} & \left(x_{11},y_{11}\right) & f_{11} \end{array}\cdots \begin{array}{cc} f_{1d-1} & f_{1d} \end{array}\\ \begin{array}{ccc} \theta_{2} & \left(x_{12},y_{12}\right) & f_{21} \end{array}\cdots \begin{array}{cc} f_{2d-1} & f_{2d} \end{array}\\ \begin{array}{ccc} \vdots & \;\vdots & \;\vdots \; \end{array}\vdots \;\vdots \;\vdots \\ \begin{array}{ccc} \theta_{N} & \left(x_{Nd},y_{Nd}\right) & f_{N1} \end{array}\cdots \begin{array}{cc} f_{Nd-1} & f_{Nd} \end{array} \end{array}\right)$$

where $\left(x_{Nd},y_{Nd}\right)$ indicates the location of a feature point, and $f_{Nd}$ represents the feature vector information, which is denoted by a binary value. d indicates the nth feature vector. For $\theta_{N}$, *N* represents the total number of angle rotation orientations (bin) formulated as:

(18) $$N=360^{{}^{\circ}}/\theta ,$$

where θ is the angle of rotation for each rotation. For example, if each rotation involves a rotation angle of 90°, then θ=90; thus, N=4, indicating four sets of angle rotation orientations. The main purpose of the voting mechanism and lookup table method is that, instead of comparing and matching every feature point, some feature points are selected and matched under various rotation angles, and the matching results are inputted into a voting mechanism. Subsequently, a lookup table is used to identify the rotation angle combination with the highest number of votes, which most likely reflects the angle difference between the sample image and the database image. Finally, this angle combination is applied to match all feature points to obtain the final matching outcome. The FBRLF rotates each feature at various angles. In other words, each angle involves the identical number and characteristics of feature vectors. Consequently, under any angle, a specific percentage of features can be randomly selected to conduct the voting mechanism using Equation (19):

(19) $$TN_{\theta_{i}}=TF\times (V/100),$$

where TF represents the total number of feature vectors, and *V* indicates the percentage ratio of features used as the votes of the voting mechanism. $TN_{\theta_{i}}$ yields the total number of features randomly selected for each angle; for $\theta_{i}$, i represents the total number of angle rotation orientations (bin). An experiment was conducted to determine the optimum percentage ratio of features randomly selected for conducting the voting mechanism. Specifically, 300 natural grayscale images were employed, among which 100 and 200 images were testing and database images, respectively. The size of each image was 800 × 600, and each of the 300 images might exhibit rotation, scaling, affine, or lighting transformation. For every testing image inputted for comparison, 100 database images similar to, and a remaining 100 database images dissimilar to this testing image are involved in the comparison. Using the procedure mentioned in Section 2.1.1 revealed that the optimum lower bound and upper bound parameters were 765 and 893, respectively. The Hamming distance threshold value was set at 40 to conduct this experiment. Figure 15 presents the experimental results.

As it can be seen that selecting 40% of the total feature points can yield a matching rate that is simply 0.1% lower than that of acquiring 100% of the total feature points. Consequently, this study adopts 40% feature points for applying the voting mechanism.

In this paragraph, the process of the voting mechanism is briefly elucidated. The following assumptions are made: A total of 100 feature vectors are identified in each image, *k* = 90, and 40% of the features are randomly selected to conduct feature matching under various rotation angles. According to these assumptions, substituting the values into Equation (19) determines that 40 features must be randomly selected from the 100 features to apply the voting mechanism. Next, these 40 features are compared with the database features under various rotation angles using Equation (10).

**Figure 16:**
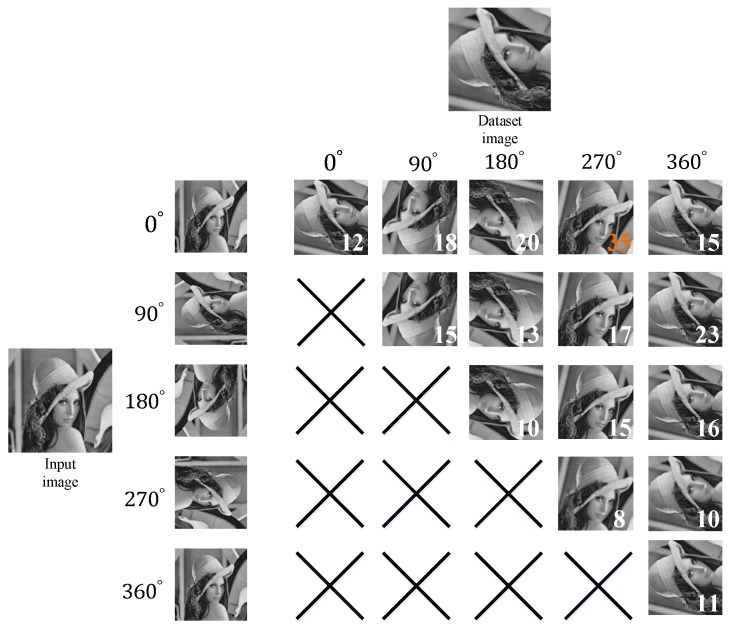
Illustration of the voting lookup table mechanism.

Table 4 illustrates the voting lookup table mechanism. This table records the voting results of comparing the 40 input features with the database features under various rotation angles. The accumulated matching outcome set is denoted as $TP=\left\{TP_{\left(0{}^{\circ},0{}^{\circ}\right)},\;TP_{\left(0{}^{\circ},90{}^{\circ}\right)},\;\ldots ,TP_{\left(\theta_{1},\theta_{2}\right)}\right\}$, where $TP_{\left(\theta_{1},\theta_{2}\right)}$ represents the accumulated matching outcome when the rotation angle $\left(\theta_{1},\theta_{2}\right)$ for the input image and database image with a rotation angle, respectively. The results show that the highest vote $TP_{MAX}$ count occurs when the angles of the input and database images are set at 0° and 270°, respectively, indicating that the input and database images are highly correlated. Subsequently, all of the input feature vectors are compared to the database feature vectors with a 270° rotation. In Table 4, the mark “X” indicates repeated angle calculations, which are omitted. In addition, a graphical example is used to illustrate the aforementioned procedure as shown in Figure 16. As a result, when an input image at 0° and the database image at 270°, it receives the highest vote count in the voting lookup table. However, if the highest vote count is lower than a specific threshold value, the input and database images are not correlated. Thus, the subsequent comparison is omitted as:

(20) $$S=\{\begin{array}{cc} 1 & if\;TP_{MAX}>\left(TN_{\theta_{i}}\times 0.8\right);\\ 0 & otherwise, \end{array}$$

where S reflects the correlation between the input and database images. S = 1 indicates that the images are highly correlated; otherwise, S = 0. $TP_{MAX}$ is the maximum value in set TP. S is evaluated on the basis of whether the highest vote count is higher than 80% of the actual vote count. If the highest vote count is less than 80% of the actual vote count, then the subsequent comparison is omitted.

**Figure 17:**
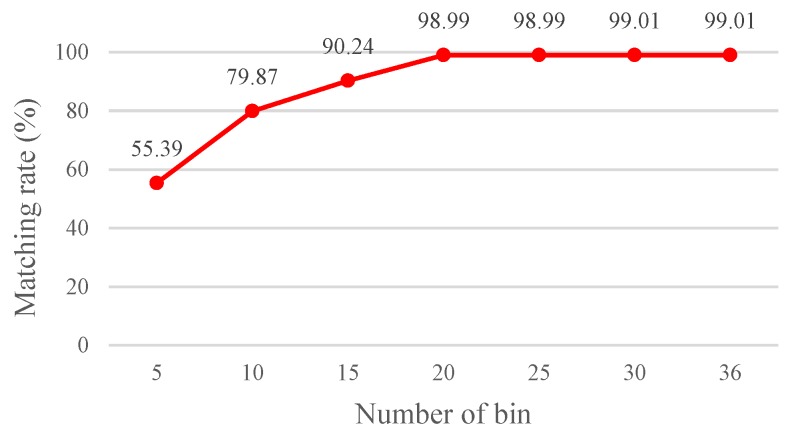
Matching rate achieved through various numbers of rotations.

**Figure 18:**
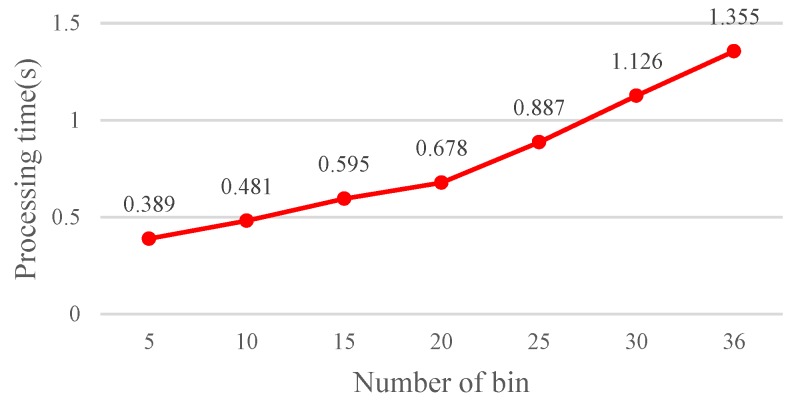
Computation time required for various numbers of rotations.

Increasing the number of angle rotation orientations (*N*) facilitates enhancement of the matching accuracy because fine angle differentiation enables determining the most fitting angle difference between two images. However, increasing the number of angle rotation orientations also increases the computation time as it can be seen in Figure 17 and Figure 18.

**Figure 19:**
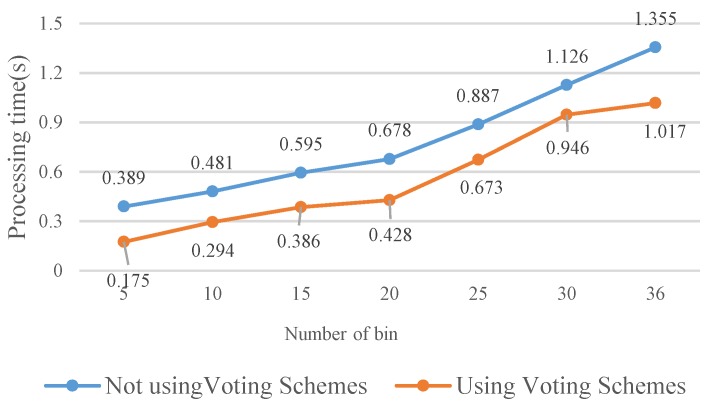
Calculation time required for the FBRLF when the voting mechanism and lookup table method are applied.

The results show that the matching rate is proportional to the number of rotations; yet, the computation time also increases. Specifically, when the number of rotations is 20 (18°/rotation), the matching rate is 98.99%. Although the matching rate further increases to 99.01% as the number of rotations is 30 or 36, the processing time increases as well. To achieve balanced performance, this study configures the number of rotations at 20. If the FBRLF is not integrated with the voting mechanism and lookup table method, then the efficiency of the overall calculation is severely reduced. For example, a feature vector with a length of 256 requires 38,400,000 (100 × 100 × 256 × 15) comparison calculations (100 input images, 100 database images, 256 sets of data in the feature vector, and 15 rotation angle combinations). However, applying the voting mechanism and lookup table method only requires 17,920,000 (40 × 100 × 256 ×15 + 100 × 100 × 256) comparison calculations. Specifically, the 40 features are adopted to apply the voting mechanism. Subsequently, if the highest vote count exceeds the threshold vote count, the corresponding rotation angle combination is used to again compare all input features with the database features. Figure 19 illustrates the calculation time of the FBRLF integrated with the voting mechanism and lookup table method. The results revealed that after this method was integrated, the time complexity of the FBRLF calculation was markedly reduced, indicating that the proposed voting mechanism can effectively reduce the search time and the overall system efficiency.

## 3. Results

This section mainly discusses related experiments conducted to test the proposed FBRLF algorithm and former fingerprint recognition algorithms. Under various photographing conditions, the feature detection matching outcomes acquired using the FBRLF algorithm were compared with those obtained using state-of-the-art local stable feature methods, namely the SIFT, SURF, KAZE, A-KAZE, BRIEF, ORB, BRISK, FREAK, and MROGH.

This study applied the well-known local descriptor assessment method to the database collected by K. Mikolajczyk and C. Schmid [34] and Oxford data set [35] to compare the FBRLF algorithm against former local feature algorithms. The first image of each type of algorithm was used as the standard image, and the testing platform was as follows. CPU: Intel i5-3320M at 2.6 GHz; random access memory (RAM): 16G DDR3; operating system: Windows 7 Service Pack 1; C language programing software: Microsoft Visual Studio 2013. The methods of SIFT, SURF, KAZE, A-KAZE, BRIEF, ORB, BRISK and FREAK are available on the V3.0 version of OpenCV framework and the official MROGH [7] method is available in GitHub. In addition, the optimal parameter list of the FBRLF used in this section as shown in Table 5, in which the term “Auto” indicates the program automatically adjusts to the optimized value.

**Figure 20:**
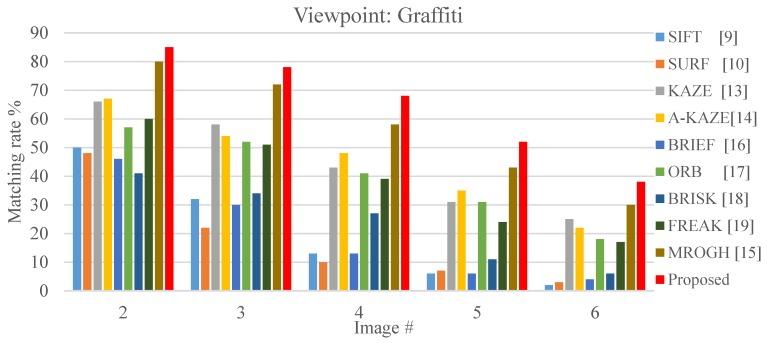
Local stable feature matching outcomes under various view-angles.

The results revealed that when view-angle variation is small, the floating-point descriptors, SIFT and SURF, resulted in matching rates greater than 40%, and the KAZE, A-KAZE and MROGH achieve matching rate greater than 70% as it can be seen in Figure 20. Among the algorithms that apply the binary matching scheme, the effect of the FBRLF is more favorable than that of the ORB, BRIEF, FREAK, and BRISK. When view-angle variation increases, the matching rate of all algorithms decreases. Because the FBRLF can fixate the rotation angle and stimulate affine features, its matching rate is more favorable than that of the MROGH under view-angle variation.

**Figure 21:**
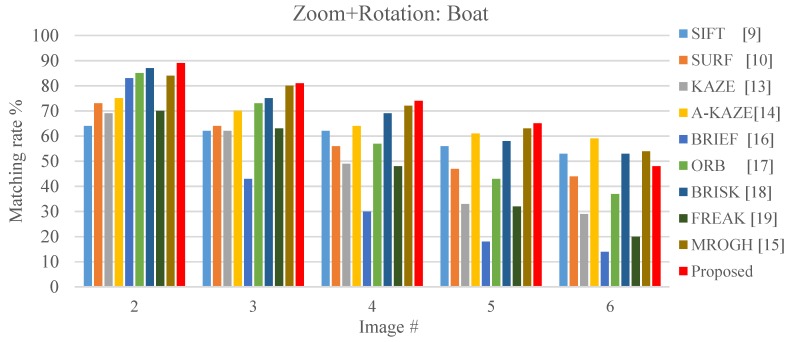
Local stable feature matching outcomes under various scales and rotation angles.

Under small scaling and rotation variation, the SIFT, SURF, and KAZE achieve matching rates greater than 60%. However, the matching rate decreases with increasing scaling and rotation variation. The proposed FBRLF can detect stable features in scale space and applied affine feature matrices; thus, the proposed algorithm exhibited superior scaling-invariant and rotation-invariant capabilities than did other algorithms as it can be seen in Figure 21.

**Figure 22:**
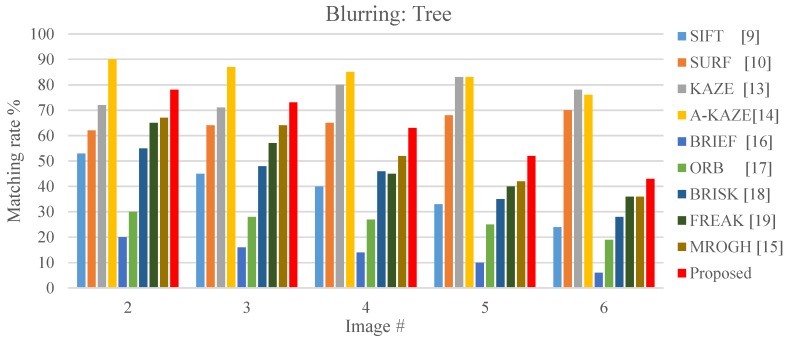
Local stable feature matching outcomes under various blur levels.

Under blurring variation, the KAZE and A-KAZE achieve matching rates greater than 70% as it can be seen from Figure 22. Among the algorithms that apply the binary matching scheme, the FBRLF is more desirable than that of the ORB, BRIEF, FREAK and BRISK. Although the feature details are lost when the testing images became blurry, the FBRLF algorithm can exhibit a higher matching rate than do the other binary match algorithms because it can identify more stable feature points.

**Figure 23:**
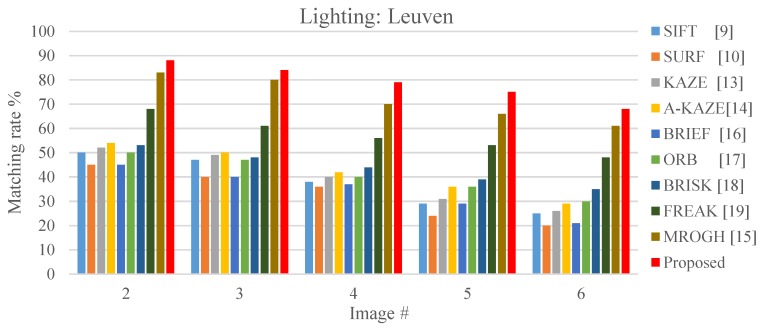
Local stable feature matching outcomes under various lighting levels.

Lighting variation refers to how the details of an object are gradually lost as it is photographed under various lighting conditions. Variation in lighting can enhance certain features in an image, while weakening other features. As it can be seen from Figure 23, the FBRIEF method can exhibit a favorable lighting-invariant capability compared to other algorithms.

**Figure 24:**
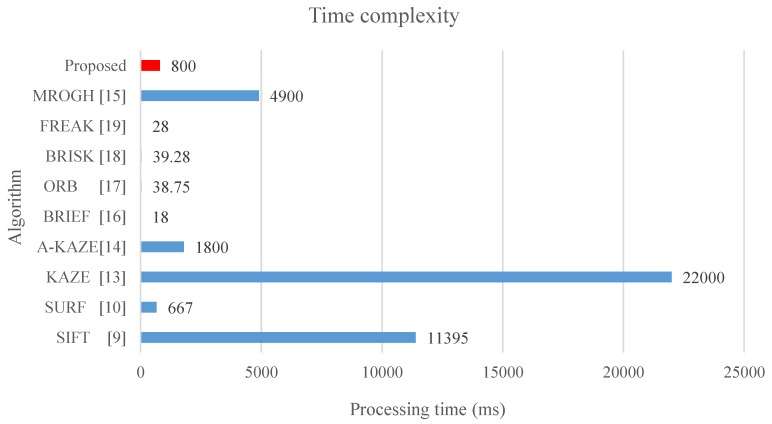
Average processing time of various local stable feature matching algorithms.

In this study, processing time refers to the time required for extracting, describing, and matching stable features among images. Moreover, the computation time is calculated with images of size 800 × 600, which are from [34], in which approximately 1500 keypoints are detected per image and the results correspond to the description and matching of all the keypoints. The results show that the BRIEF exhibits the shortest processing time among all the algorithms as it can be seen from Figure 24, because it only involves comparing the coordinates of the feature points, which are not scale-invariant and rotation-invariant. The ORB adopts the exhaustive method to identify correlated random points, and the BRISK applies various Gaussian core models to effectively control image noise; thus, these two methods also incur additional processing time. Otherwise, the FREAK proposes to apply the saccadic search by parsing their descriptors in the matching process, and thus it decreases the matching time. Although the methods with binary descriptors can achieve processing time shorter than 40 milliseconds, the matching performance is inferior to other binary descriptor methods such as MROGH and FBRLF. The SIFT and SURF have high time complexity because they generate linear Gaussian blur pyramids. However, the SURF adopted integral images to resolve problems on the pyramids and feature vectors, and thus effectively reduces the time complexity. The KAZE and A-KAZE exhibit excessively high complexity because they involve nonlinear image pyramids computation. Moreover, the MROGH provides good quality descriptors, but it is still with long computation time. In contrast, the proposed FBRLF algorithm exhibits not only scale-invariant, rotation-invariant, view-angle-invariant, and lighting-invariant capabilities, but also yields high computational efficiency.

To verify the performance of the proposed descriptor in the large-scale testing, the additional database, Oxford data set [35], was included to further verify the effectiveness of the proposed algorithm against other local stable feature methods. This study follows the evaluation procedure in [34] to present the recall vs 1-precision curve, and its relevant equations are as follows.

(21) $$\mathrm{recall}=\frac{number\;of\;correct\;matches}{number\;of\;correspondence}$$

(22) $$1-\mathrm{precision}=\frac{number\;of\;false\;matches}{number\;of\;matches}$$

**Figure 25:**
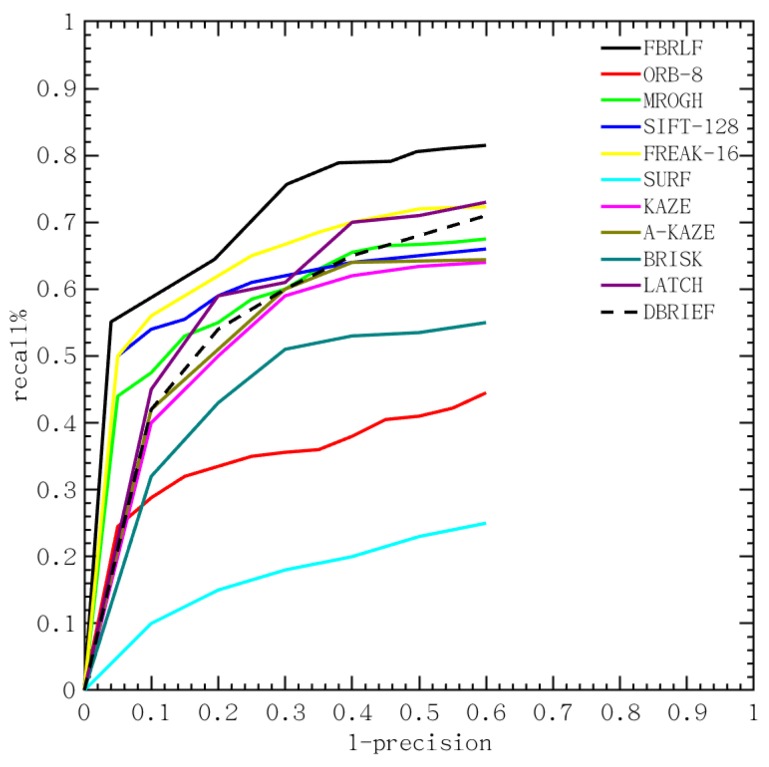
Average precision-recall curves for ORB, MROGH, SIFT, SURF, KAZE, A-KAZE, FREAK, BRISK, LATCH, DBRIEF and the proposed FBRLF on the Oxford data set.

As illustrated in Figure 25, the proposed FBRLF algorithm performs the best in all types of the Oxford data set, such as Graffiti, Boat, Wall and so on. Notably, the FREAK, BRIEF, ORB and BRISK are faster than the proposed FBRLF, but the proposed FBRLF can achieve the highest precision-recall curve than that of those methods. The proposed FBRLF also can achieve better performance than the descriptor learning methods, including LATCH and DBRIEF. In addition, although FBRLF is slower than that of the ORB and FREAK in the description phase, the FBRLF can obtain the best precision-recall curve than those methods as it can be seen from Table 6. The FBRLF method has utilized rotation-invariant features by fixating feature point rotation at various angles, and consequently leaded slightly increasing matching time than that of the ORB. Meanwhile, the FBRLF method requires the lowest storage requirement as that of the ORB. Finally, the proposed FBRLF method not only demonstrates good precision-recall performance, but also efficient on computation and storage.

## 4. Discussion and Conclusions

The contributions of the FBRLF algorithm proposed in this study are described as follows. (1) A scale space during feature detection was established using a single threshold value to detect whether features are undesirable because stable features cannot be detected through decimating the image pyramid. Consequently, the MAGAST feature detection method is proposed to adopt a dynamic threshold value to identify representative features. The experiments of this study also verified that this method effectively increased the repeatability of feature points. (2) The feature description technique of the proposed algorithm involves using a Gaussian template and integral images to efficiently calculate the effect of neighboring pixels on feature points, and different Gaussian core models were applied to statically process the neighboring pixels, thus alleviating the problem of high computation cost. The experimental results confirmed that this feature description technique effectively resolved the problem of time complexity and controlled neighboring noise signals. (3) The voting mechanism and lookup table method were integrated with the proposed algorithm to reduce the time cost of the feature matching. Conventional feature matching methods mostly adopt the exhaustive or indexing methods. The exhaustive method incurs extra processing time when the number of features increases, and the indexing method also requires high temporal and calculation costs when establishing an index structure. According to the experimental data, this study shows that the voting mechanism and lookup table technique not only reduce the processing time of the matching, but also effectively enhance the feature point matching rate. The experimental results confirm that the proposed algorithm can achieve a superior matching performance than that of the state-of-the-art binary descriptors (ORB, BRISK, and FREAK), and the processing time and storage requirement are also lower than those of the floating-point descriptors (SIFT, SURF, and MROGH). Finally, the FBRLF source code is available in [36].

## Figures and Tables

**Table 1:** Illustration of the MAGAST feature detection.

Line	Code	Step1	Step2	Step3
03	**WHILE** (Score < Lower Bound || Score > Upper Bound)	Ture	Ture	Ture
04	Score = use Threshold to Detect AGAST features on image	500	250	180
05	**IF** (Lower Bound > Score)	False	False	False
12	Left = Threshold	4	6	7
13	Threshold = (Threshold + Right) >>1	6	7	7
14	**IF** (Left == Threshold)	False	False	Ture
18	**END**	False	False	Ture

**Table 2:** Six groups of images with various sizes.

Group	#1	#2	#3
Image Size	160×120	320×240	640×480
Group	#4	#5	#6
Image Size	800×600	1024×768	1280×960

**Table 3:** Lower and upper bound parameters of each image group.

Group	#1	#2	#3
Lower Bound	8	235	354
Upper Bound	198	369	686
Group	#4	#5	#6
Lower Bound	765	931	1367
Upper Bound	893	1267	1593

**Table 4:** Voting lookup table mechanism.

Voting Mechanism Lookup Table		Database Image				
		0°	90°	180°	270°	360°
**Input Image**	0°	12	18	20	35	15
	90°	-	15	13	17	23
	180°	-		10	15	16
	270°	-	-	-	8	10
	360°	-	-	-	-	11

**Table 5:** Parameters of the Fast Binary Robust Local Feature (FBRLF) algorithm.

Section	Parameter	Value	Description
2.1	*K*	1.6	Scale ratio
2.1	*Left*	0	Left shift operator (Initial value)
2.1	*Right*	255	Right shift operator (Initial value)
2.1	*T*	128	Unadjusted threshold value (Initial value)
2.1	$T^{\prime }$	Auto	Adjusted threshold value
2.1	*Score*	Auto	Score of feature points
2.1	*n*	3	Scale space
2.1.1	*Lower Bound*	Auto	Depend on image size
2.1.1	*Upper Bound*	Auto	Depend on image size
2.4	$f_{n_{d}}$	256	Number of keypoint descriptor
2.5.1	$T_{HD}$	40	Hamming distance threshold value
2.5.2	*N*	20	Number of angle rotation orientations (bin)
2.5.2	*V*	40	Percentage ratio of features

**Table 6:** Computation costs and storage requirements of different methods of a single descriptor.

	SIFT	SURF	MROGH	ORB	FREAK	FBRLF
Description (in ms)	2.65	1.65	4.83	0.019	0.025	0.1197
Matching (in ns)	1072	843	1567	16	31	21
Storage (in bytes)	128*4	64*4	192*4	32	64	32
